# CCN3/NOV as a potential therapeutic target for diverticular disease: A proteome-wide Mendelian randomization study

**DOI:** 10.1097/MD.0000000000040467

**Published:** 2024-11-08

**Authors:** Masahiro Yoshikawa, Kensuke Asaba

**Affiliations:** a Division of Laboratory Medicine, Department of Pathology and Microbiology, Nihon University School of Medicine, Tokyo, Japan; b Department of Computational Diagnostic Radiology and Preventive Medicine, The University of Tokyo Hospital, Tokyo, Japan.

**Keywords:** Bayesian colocalization, diverticular disease, genome-wide association study, Mendelian randomization, protein quantitative trait loci

## Abstract

Genome-wide association studies (GWAS) identified over 100 susceptibility loci and candidate causal genes for diverticular disease (DD) at the transcriptional level. However, effective therapeutics or preventions based on underlying disease mechanisms remain to be elucidated. In this study, we explored potential causal genes for DD at the protein level. We used 2 GWAS summary statistics of DD; 1 was obtained from the United Kingdom Biobank (UKBB) with 31,917 cases and 419,135 controls, and the other from the FinnGen consortium with 30,649 cases and 301,931 controls. For the primary analysis, we employed proteome-wide Mendelian randomization (MR) studies using 738 cis-acting protein quantitative trait loci (pQTLs) for 735 plasma proteins from the 5 published studies. For external validation, we conducted 2-sample MR analyses using plasma pQTLs of the screened proteins from another study by deCODE genetics. Moreover, we performed a series of sensitivity analyses including reverse MR and Bayesian colocalization tests. The primary MR identified 4 plasma proteins that were associated with DD risk including CCN3/NOV (odds ratio [OR], 0.98; 95% confidence interval [CI], 0.97–0.99; *P* = 1.2 × 10^−11^ for UKBB. OR, 0.73; 95% CI, 0.66–0.81; *P* = 7.2 × 10^−10^ for FinnGen). The validation MR well replicated the primary result of CCN3/NOV (OR, 0.95; 95% CI, 0.93–0.96; *P* = 1.9 × 10^−11^ for UKBB. OR, 0.43; 95% CI, 0.33–0.56; *P* = 7.0 × 10^−10^ for FinnGen). Sensitivity analyses supported the causal association. We prioritized plasma CCN3/NOV protein as a protective factor for DD for follow-up functional studies to elucidate the disease mechanisms and therapeutics.

## 1. Introduction

Diverticular disease (DD) of the intestine is a common condition marked by the presence of multiple sacs or pouches at weak points in the muscle layers of the bowel wall.^[1]^ The prevalence of DD is <10% in individuals younger than 40 years old and more than 50% in those older than 80 years old;^[2]^ however, the incidence is currently increasing in younger groups.^[1]^ Most patients may be asymptomatic, but 10% to 25% of cases develop complications such as diverticulitis, hemorrhage, fistula formation, and perforation,^[1,3]^ which require endoscopic or surgical therapies and can be a high burden. In addition to environmental factors, genetic predisposition is supposed to play an essential role in the pathogenesis of disease.^[1]^ To date, genome-wide association studies (GWAS) have reported more than 100 genome-wide significant loci and candidate causal genes in the European population, inferring potential mechanisms of DD onset, such as dysregulation of wall structure, mortality, mucus, and membrane transport.^[4–8]^ However, these studies were conducted at the transcriptional level. Effective therapies or preventions based on underlying disease mechanisms remain to be established.

The proteome is the central layer between the genome and phenome.^[9]^ Proteins play key roles in diverse biological processes in the human body, including cellular signaling, membrane transport, tissue growth and repair, and infection defense.^[10]^ Therefore, the proteome and proteins represent an important source of druggable targets for disease. Recently, many GWAS of levels of plasma proteins have identified large number of single nucleotide polymorphisms (SNPs) that are significantly associated with protein expression (i.e., protein quantitative trait loci, or pQTLs).^[11]^ Using the summary statistics of these pQTL studies, a proteome-wide Mendelian randomization (MR) analysis allows researchers to systematically examine the causal effects of plasma proteins on a human phenotype of interest.^[12,13]^ In this study, we comprehensively examined causal associations between hundreds of plasma proteins and DD using a proteome-wide MR approach, followed by a series of validation and sensitivity analyses including reverse MR and Bayesian colocalization tests. Consequently, we prioritized plasma CCN3 (cellular communication network factor 3, also known as nephroblastoma overexpressed, or NOV) protein as a potential therapeutic target for DD.

## 2. Materials and methods

### 2.1. Data sources

The SNPs as genetic instrument variables (IVs) for levels of plasma proteins were available from the published study by Zheng et al.^[12]^ The selection and validation criteria of the IV SNPs were described in the original paper. Briefly, they collected 3606 pQTLs for 2656 proteins from 5 published GWAS in the European population by Sun et al,^[10]^ Suhre et al,^[14]^ Folkersen et al,^[15]^ Yao et al,^[16]^ and Emilsson et al ^[17]^ and used the following criteria: (1) they selected pQTLs associated with any protein with *P* < 5.0 × 10^−8^; (2) they removed pQTLs and proteins encoded by genes within the major histocompatibility complex region because of the complex linkage disequilibrium (LD) structure; (3) they clumped IV SNPs at r^2^ < 0.001 to include independent pQTLs for each other; (4) they excluded pQTLs associated with more than 5 proteins due to potential pleiotropy; and (5) they selected consistent pQTLs that exhibited no heterogeneity in SNP effects across the 5 studies. As a result, 1064 pQTLs of 955 proteins were selected. Among them, we used only 738 cis-acting pQTLs (within 500 kb from the leading pQTL of the tested protein) for 735 plasma proteins in the present study (Table S1, Supplemental Digital Content, http://links.lww.com/MD/N876). Another plasma pQTL dataset was obtained from the GWAS by Ferkingstad et al ^[18]^ and the deCODE genetics (4907 plasma proteins from 35,559 Icelanders) (https://www.decode.com/summarydata/).

For the datasets of genetically predicted DD risk, we used GWAS summary statistics from the published study using the United Kingdom Biobank (UKBB) by Schafmayer et al.^[7]^ The dataset included 31,917 cases (International Classification of Diseases (ICD)-10 code K57 and ICD-9 code 562) and 419,135 controls and was available from the GWAS Catalog^[19]^ (http://ftp.ebi.ac.uk/pub/databases/gwas/summary_statistics/GCST008001-GCST009000/GCST008105/). Another GWAS dataset was retrieved from the FinnGen consortium^[20]^ Data Freeze 11 with 37,886 cases (ICD-10 code K57, ICD-9 code 562, and ICD-8 code 562) and 360,393 controls (https://r11.finngen.fi/pheno/K11_DIVERTIC).

There was no apparent sample overlap that might lead to substantial bias among these datasets.^[21]^

### 2.2. Study design

The study design is summarized in Table 1.

**Table 1:** Study design.

Primary analysis	External validation
*Methods*	
Proteome-wide Mendelian randomization (comprehensive screening)	Two-sample Mendelian randomization
	Reverse Mendelian randomization
	Bayesian colocalization
*Proteins*	
738 cis-pQTLs for 735 plasma proteins by Zheng et al from 5 published studies	cis-pQTLs for the screened proteins from the deCODE genetics by Ferkingstad et al
*Diverticular disease*	
UK Biobank GWAS by Schafmayer et al (31,917 cases and 419,135 controls)	
FinnGen GWAS Data Freeze 11 (37,886cases and 360,393 controls)	

Abbreviations: GWAS = genome-wide association study, pQTLs = protein quantitative trait loci.

For the primary analysis, we employed proteome-wide MR studies and screened plasma proteins comprehensively that were associated with the risk of DD using the 738 cis-pQTLs for 735 proteins as an exposure dataset and the 2 GWAS summary statistics of DD as outcome datasets. For external validation, we performed 2-sample MR analyses using the plasma pQTL summary statistics obtained from the deCODE genetics as exposure datasets and the 2 GWAS summary statistics as outcome datasets to validate the proteins that were screened by the primary analysis. We also performed reverse MR analyses using the 2 GWAS summary statistics of DD as exposure datasets and the deCODE pQTL summary statistics as outcome datasets to investigate reverse causality. Moreover, we conducted Bayesian colocalization (Coloc) tests to assess whether the plasma proteins and DD shared the same causal variants using the deCODE pQTL summary statistics.

The study followed the STROBE guidelines.^[22]^

In this study, we used only publicly available summary-level datasets. Ethical approval and consent to participate were obtained in the original studies.

### 2.3. Mendelian randomization

All MR analyses were conducted using the TwoSampleMR package (version 0.5.6) in R software (version 4.0.3). The functions were described in the following website; https://mrcieu.github.io/TwoSampleMR/reference/index.html. Two-sample MR analysis must satisfy the following 3 IV assumptions: (1) the IV SNP is associated with the exposure; (2) the IV SNP is associated with the outcome only through the exposure; and (3) the IV SNP is not associated with any confounders.^[23]^ To satisfy IV assumption 1, we selected SNPs as IVs that were associated with exposures with *P* < 5.0 × 10^−8^. We clumped them (r^2^ < 0.001 and 10,000 kb distance) using the clump_data function to include independent IV SNPs that were not in LD with the other SNPs. We excluded palindromic SNPs with a minor allele frequency (MAF) > 0.42. We evaluated the IV strength by calculating R^2^ (proportion of phenotypic variance explained by each SNP) and the *F*-statistic.^[24]^ SNPs with an *F*-statistic below 10 were considered weak instruments.^[25]^ We also employed the Steiger filtering method using the steiger_filtering function to examine whether the R^2^ was significantly larger in exposure than in outcome (*P* value < 0.05) and infer the causal direction of each SNP.^[26]^ In addition, we searched for pQTLs that were significantly associated with possible pleiotropic effects on DD (*P* < 5.0 × 10^−8^) and possibly violated the IV assumptions by use of the GWAS Catalog.^[19]^ We extracted summary statistics for each IV SNP from the outcome GWAS summary statistics and harmonized them using the harmonise_data function. The Wald ratio calculated an MR estimate (Beta) that was equivalent to the log_e_ odds ratio (OR) of DD per standard deviation increase in plasma protein levels when only a single IV SNP was available.^[23]^ When 2 or more IV SNPs were available, the inverse variance weighted (IVW) method meta-analyzed each MR estimate of the Wald ratio using the mr function.^[23]^ We calculated Cochrane *Q* statistic using the mr_heterogeneity function, which measured heterogeneity among the causal estimates across all IV SNPs.^[23]^ When heterogeneity was detected (*P* value < 0.05) in the IVW method, the multiple random-effect (MRE) model was used.^[27]^ Otherwise, the fix-effect (FE) model was used. In the 2-sample MR analyses for external validation, we selected only cis-acting pQTLs as IV SNPs, which located in the vicinity of the encoding gene (within ± 500 kb from the leading pQTL of the tested protein) and were independent (r^2^ < 0.001). The leading pQTL was defined as the SNP that was associated with the tested protein with the smallest *P* value. Bonferroni correction was applied for multiple testing and a *P* value below 6.8 × 10^−5^ (0.05/735 proteins) was considered statistically significant in the MR results.

### 2.4. Reverse MR

To investigate reverse causality, the 2 GWAS of DD were used as exposure datasets and the deCODE pQTLs were used as outcome datasets. We selected IV SNPs that were significant (*P* < 5.0 × 10^−8^) and independent (r^2^ < 0.001), and excluded ambiguous palindromic SNPs (MAF > 0.42). We employed the IVW method as a main analysis as well as the MR-Egger regression method, the weighted median method, and the MR-PRESSO (Pleiotropy Residual Sum and Outlier) outlier test using the run_mr_presso function as sensitivity analyses. For the IVW method, the MRE or FE model was selected in the same way as the forward MR. In the MR-Egger regression method, the significant nonzero intercept estimates the magnitude of horizontal pleiotropy.^[28]^ The weighted median method provides a valid estimate when only more than half of the IV SNPs satisfy IV assumptions.^[23,28]^ The MR-PRESSO outlier test corrects horizontal pleiotropy by removing outlier SNPs.^[29]^ A *P* value below 0.05 was considered statistically significant in the reverse MR analyses.

### 2.5. Bayesian colocalization test

To investigate whether the plasma proteins and DD risk shared the same causal SNPs, the Coloc test^[30]^ was conducted by the coloc package (version 5.2.3) in R software (version 4.0.3). We used the deCODE pQTLs and the 2 GWAS summary statistics of DD in cis regions (within ± 500 kb from the leading pQTL of the tested protein) as described above. If duplicated SNPs were found in pQTL or GWAS datasets, the SNP with the smallest *P* value was included. pQTL and GWAS SNPs were merged by identification numbers (rsID) using the merge function. We calculated the posterior probabilities (PPs) of the 5 hypotheses using the coloc.abf function: H0, no association with either protein expression or disease; H1, association with protein expression, not with disease, H2, association with disease, not with protein expression, H3, association with protein expression and disease by independent SNPs, H4; association with both protein expression and disease by shared causal SNPs. A large PP of H4 (PP.H4) above 0.80 strongly supported causal SNPs shared by both protein expression and disease. A prior probability of 1 × 10^−4^ for H1 and H2 and a prior probability of 1 × 10^−5^ for H4 were assigned as the default settings. The locus compare plots were visualized using the locuscompareR package in R software.^[31]^

## 3. Results

### 3.1. Mendelian randomization

**Figure 1.**
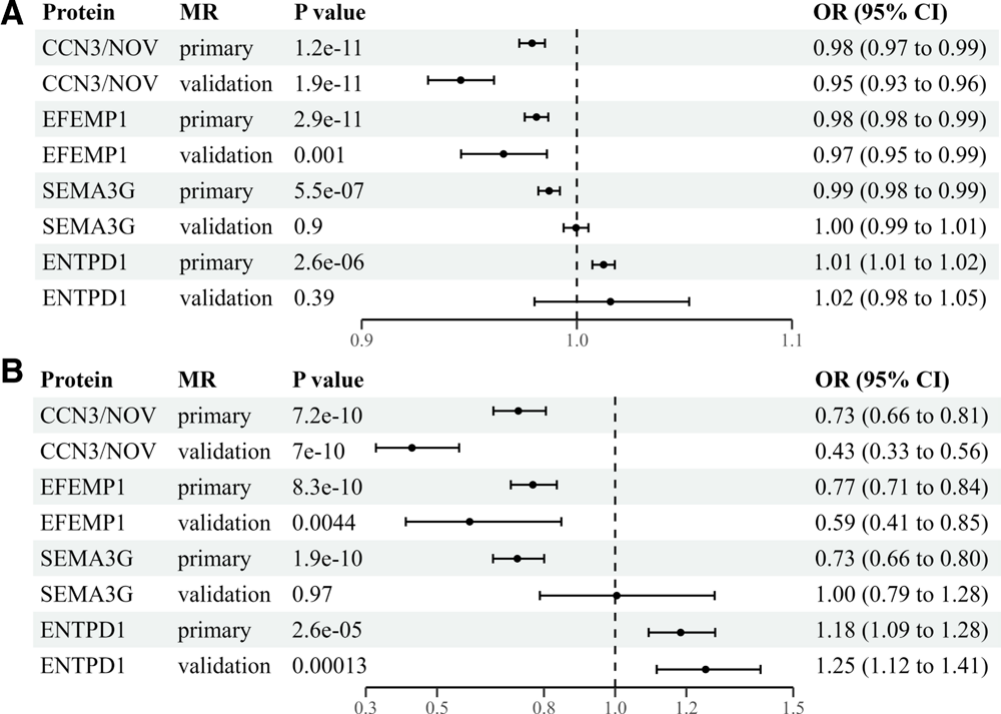
Forrest plots for the Mendelian randomization (MR) results of the causal effects of plasma proteins on diverticular disease (DD) risk in the primary and validation analyses using (A) UK Biobank and (B) FinnGen genome-wide association study summary statistics. The odds ratio (OR) and 95% confidence interval (CI) for the risk of DD is presented as per standard deviation increase in plasma protein levels.

The overall MR findings are presented in Table 2 and Figure 1. The characteristics of IV SNPs are presented in Table S2, Supplemental Digital Content, http://links.lww.com/MD/N876

**Table 2:** Mendelian randomization results using protein quantitative trait loci as exposures and genome-wide association study of diverticular disease as outcomes.

Exposure protein	MR	SNP	Method	Outcome 1 (UKBB)			Outcome 2 (FinnGen)		
				Beta	SE	*P* value	Beta	SE	*P* value
CCN3/NOV	Primary	rs58936256	Wald ratio	−0.021	0.003	**1.2 × 10^−11^**	−0.317	0.051	**7.2 × 10^−10^**
CCN3/NOV	Validation	rs11779998	Wald ratio	−0.055	0.008	**1.9 × 10^−11^**	−0.845	0.137	**7.0 × 10^−10^**
EFEMP1	Primary	rs3791679	Wald ratio	−0.019	0.003	**2.9 × 10^−11^**	−0.263	0.043	**8.3 × 10^−10^**
EFEMP1	Validation	rs9309272, rs3791663	IVW	−0.035	0.011	.0010	−0.525	0.184	.0044
SEMA3G	Primary	rs2016575	Wald ratio	−0.013	0.003	**5.5 × 10^−7^**	−0.321	0.050	**1.9 × 10^−10^**
SEMA3G	Validation	rs13091025	Wald ratio	−0.0004	0.003	.90			
		rs13091025, rs115483359	IVW				0.004	0.123	.97
ENTPD1	Primary	rs11188501	Wald ratio	0.012	0.003	**2.6 × 10^−6^**	0.169	0.040	**2.6 × 10^−5^**
ENTPD1	Validation	rs28378465, rs189315256	IVW	0.016	0.018	.39	0.226	0.059	1.3 × 10^−4^

A bold *P* value was considered statistically significant (*P* < 6.8 × 10^−5^).

Abbreviations: GWAS = genome-wide association study, IVW = inverse variance weighted, MR = Mendelian randomization, pQTLs = protein quantitative trait loci, SE = standard error, SNP = single nucleotide polymorphism, UKBB = UK Biobank.

**Figure 2.**
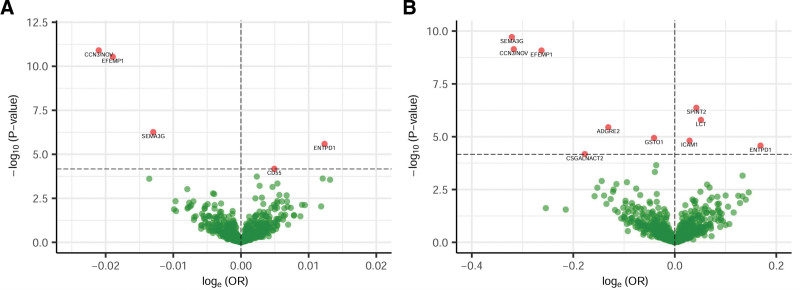
Volcano plots for the primary proteome-wide Mendelian randomization results of the causal effects of 735 plasma proteins on diverticular disease (DD) risk using (A) UK Biobank and (B) FinnGen genome-wide association study summary statistics. The odds ratio (OR) for the risk of DD is presented as per standard deviation increase in plasma protein levels. The horizontal dashed line corresponds to *P* = 6.8 × 10^−5^.

The primary proteome-wide MR analyses using the UKBB and FinnGen GWAS summary statistics commonly prioritized 4 plasma protein levels that were significantly associated with DD risk (Fig. 2), including CCN3/NOV (OR, 0.98; 95% confidence interval [CI], 0.97 to 0.99; *P* = 1.2 × 10^−11^ for UKBB. OR, 0.73; 95% CI, 0.66 to 0.81; *P* = 7.2 × 10^−10^ for FinnGen), EFEMP1 (OR, 0.98; 95% CI, 0.98 to 0.99; *P* = 2.9 × 10^−11^ for UKBB. OR, 0.77; 95% CI, 0.71 to 0.84; *P* = 8.3 × 10^−10^ for FinnGen), SEMA3G (OR, 0.99; 95% CI, 0.98 to 0.99; *P* = 5.5 × 10^−7^ for UKBB. OR, 0.73; 95% CI, 0.66 to 0.80; *P* = 1.9 × 10^−10^ for FinnGen), and ENTPD1 (OR, 1.01; 95% CI, 1.01 to 1.02; *P* = 2.6 × 10^−6^ for UKBB. OR,1.18; 95% CI, 1.09 to 1.28; *P* = 2.6 × 10^−5^ for FinnGen).

To validate the primary findings, the plasma pQTL datasets for the 4 proteins were obtained from the deCODE genetics. The 2-sample MR analyses for external validation revealed that plasma CCN3/NOV protein levels were significantly associated with a decreased risk of DD (OR, 0.95; 95% CI, 0.93 to 0.96; *P* = 1.9 × 10^−11^ for UKBB. OR, 0.43; 95% CI, 0.33 to 0.56; *P* = 7.0 × 10^−10^ for FinnGen). On the other hand, the associations between the EFEMP1 protein levels and DD risk were only suggestive (*P* = .0010 for UKBB. *P* = .0044 for FinnGen). The ENTPD protein levels were not associated with DD risk for UKBB whereas they were suggestively associated with an increased risk for FinnGen (*P* = 1.3 × 10^−4^). The 2-sample MR analysis showed no association between plasma SEMA3G protein levels and DD risk (*P* > .05).

The *F*-statistic of each IV SNP was above 10 (Table S2, Supplemental Digital Content, http://links.lww.com/MD/N876), suggesting that a weak instrumental bias was unlikely. The Steiger filtering method showed that the R^2^ of each IV SNP was significantly larger in exposure than in outcome (*P* < .05) (Table S2, Supplemental Digital Content, http://links.lww.com/MD/N876), suggesting that reverse causality was unlikely. The GWAS Catalog suggested that IV SNPs for the CCN3/NOV and SEMA3G proteins were not associated with any other diseases and traits that could have an influence on a DD risk. However, IV SNPs for the EFEMP1 protein (rs3791679, rs9309272, and rs3791663) were significantly associated with the risk of inguinal hernia, which could be a complication of DD.^[32]^

### 3.2. Reverse MR

The overall findings in the reverse MR analyses and characteristics of IV SNPs are presented in Tables S3 and S4, Supplemental Digital Content http://links.lww.com/MD/N876 The *F*-statistic of each IV SNP was above 10, suggesting that a weak instrumental bias was unlikely.

When the UKBB GWAS was used as an exposure dataset, the IVW method suggested that the risk of DD was not causally associated with plasma CCN3/NOV (IVW-FE model, *P* = .11), ENTPD1 (IVW-MRE model, *P* = .86), or SEMA3G (IVW-FE model, *P* = .69) protein levels, and the other MR methods showed comparable results. However, the IVW method suggested that the risk of DD might be causally associated with plasma EFEMP1 levels (IVW-FE model, *P* = .04).

When the FinnGen GWAS was used as an exposure dataset, the IVW-MRE model suggested that DD was not causally associated with plasma CCN3/NOV (*P* = .68), EFEMP1 (*P* = .82), ENTPD1 (*P* = .41), or SEMA3G (*P* = .13) levels. The MR-Egger intercepts suggested little evidence of horizontal pleiotropy. The weighted median method and the MR-PRESSO outlier test showed comparable results except for plasma EFEMP1 levels.

### 3.3. Bayesian colocalization

**Figure 3.**
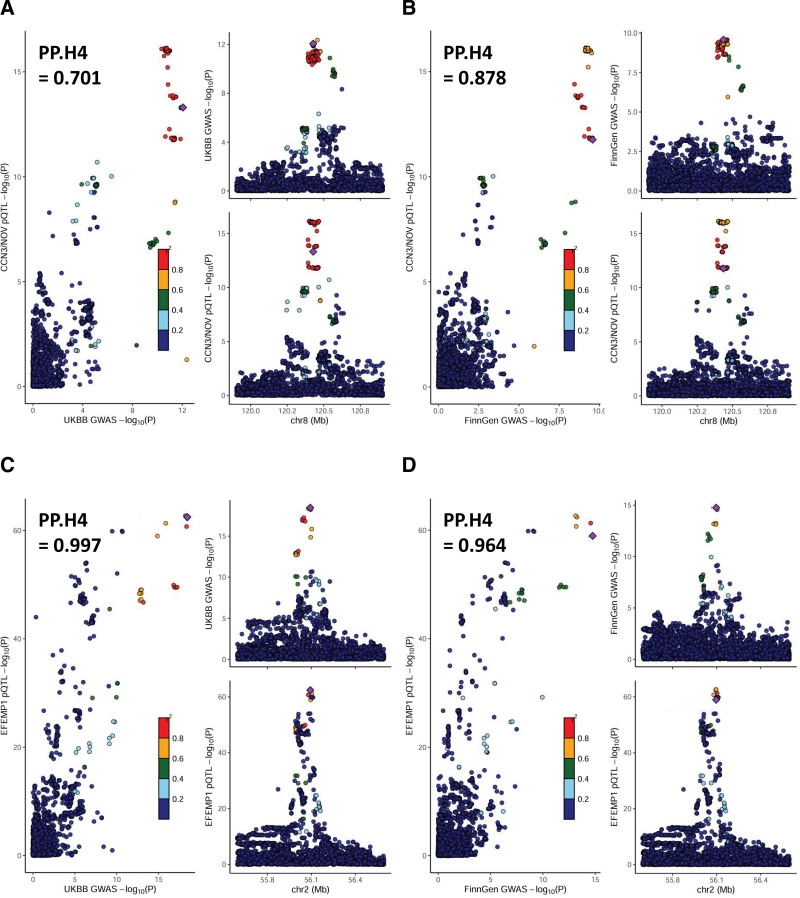
Locus compare plots for the Colocalization results of single nucleotide polymorphisms (SNPs) that were associated with plasma proteins and diverticular disease using (A) CCN3/NOV protein quantitative trait loci (pQTLs) and UK Biobank (UKBB) genome-wide association study (GWAS), (B) CCN3/NOV pQTLs and FinnGen GWAS, (C) EFEMP1 pQTLs and UKBB GWAS, (D) EFEMP1 pQTLs and FinnGen GWAS. Each dot represents an SNP whose color indicates linkage disequilibrium (*r*^2^) with the GWAS lead variant shown as a purple diamond. The chromosome (chr) and genomic position (GRCh37) are shown on the X-axis in each right panel. PP.H4 indicates a posterior probability of H4.

The Coloc results are presented in Table 3 and Figure 3.

**Table 3:** Colocalization results between deCODE protein quantitative trait loci and genome-wide association study of diverticular disease.

Protein	Diverticular disease	PP.H3	PP.H4	Number of SNPs
CCN3/NOV	UKBB	0.299	0.701	3488
CCN3/NOV	FinnGen	0.122	0.878	5250
EFEMP1	UKBB	0.003	0.997	3873
EFEMP1	FinnGen	0.036	0.964	5671
SEMA3G	UKBB	0.527	0.468	1727
SEMA3G	FinnGen	0.453	0.547	3457
ENTPD1	UKBB	0.233	0.764	3195
ENTPD1	FinnGen	1.000	0.000	4912

Abbreviations: PP of H3 = posterior probability of H3, PP.H4 = posterior probability of H4, SNP = single nucleotide polymorphism, UKBB = UK Biobank.

The PP.H4 between CCN3/NOV pQTLs and the UKBB GWAS was not strong enough but only marginal (PP.H4 = 0.701) (Fig. 3A). However, the Coloc test supported causal SNPs shared between CCN3/NOV pQTLs and the FinnGen GWAS (PP.H4 = 0.878) (Fig. 3B). Shared causal SNPs between EFEMP1 pQTLs and both the UKBB and FinnGen GWASs were also supported (PP.H4 = 0.997 and 0.964, respectively) (Fig. 3C and D). The Coloc tests did not support shared causal SNPs between ENTPD1 or SEMA3G protein levels and DD.

## 4. Discussion

An association between a single pQTL and a disease estimated by the Wald ratio does not necessarily mean causality (the protein causes the disease) but also reverse causality (the disease induces protein expression), horizontal pleiotropy (the shared causal variant affects the protein and the disease by 2 independent pathways), or confounding (the leading pQTL and the leading IV SNP of the disease are in LD).^[12,33]^ Horizontal pleiotropy and confounders can violate IV assumptions 2 and 3, respectively.^[23]^ The Coloc test can distinguish confounding from causality or horizontal pleiotropy.^[12]^ Our comprehensive proteome-wide MR analysis prioritized the 4 plasma proteins that were associated with DD risk. Among them, we highly prioritized CCN3/NOV protein as a potential protective factor for DD among the 735 plasma proteins for the following reasons: CCN3/NOV protein was validated by the 2-sample MR analysis; the Steiger filtering method and reverse MR analysis suggested that reverse causality was unlikely; and the Coloc test suggested that confounding was also unlikely although the PP.H4 was only marginal when the UKBB GWAS was used; and no pleiotropic IV SNP for CCN3/NOV protein was found using the GWAS Catalog. EFEMP1, SEMA3G, and ENTPD1 proteins were less prioritized by the present study due to possible reverse causality, lack of reproducibility, or lack of colocalization. Moreover, the IV SNPs for EFEMP1 protein were associated with inguinal hernia, which could be a complication of DD,^[32]^ suggesting that they had a pleiotropic effect.

To the best of our knowledge, this is the first study to prioritize causal genes for DD at the protein level. Five GWAS of DD^[4–8]^ reported 1, 3, 39, 48, and 142 significant loci and prioritized candidate causal genes including CCN3/NOV and EFEMP1 at the transcriptional level. The prioritized genes by GWAS indicated several potential mechanisms of DD onset including dysregulated intestinal wall structure.^[6–8]^ For example, EFEMP1 (epithelial growth factor-containing fibulin extracellular matrix protein 1) encodes an extracellular matrix (ECM) protein, and EFEMP1 knockout mice are susceptible to inguinal hernias.^[7]^ Consistently, our study suggested that, although less prioritized, EFEMP1 protein might be a protective factor for DD. Similarly, CCN3/NOV has a known role in the ECM.^6^ Studies suggest that abnormal ECM remodeling can lead to fibrosis^[34]^ and that the CCN3/NOV protein can inhibit fibrosis by promoting ECM remodeling^[35]^ in multiple tissues and diseases. In the colonic wall in DD as well, ECM remodeling and fibrosis may play an important pathogenic role.^[36,37]^ These studies may support our result that CCN3/NOV protein was causally associated with a decreased risk of DD. The CCN3/NOV protein binds to multiple cell surface receptors,^[38]^ and is detected in the circulating blood of normal subjects at a concentration of 350 to 400 ng/ml.^[39]^ CCN3/NOV expression was upregulated by inhibiting Notch signaling in mouse colonic epithelial cells.^[40]^ Taken together, these findings suggest the possibility that the CCN3/NOV protein may be a therapeutic target for multiple disorders including DD.

The present study has several major limitations. First, the Wald ratio with a single IV SNP cannot distinguish causality from pleiotropy,^[33]^ and it is difficult to strictly satisfy IV assumption 2.^[41]^ We used only cis-acting pQTLs as IVs because trans-acting pQTLs were more likely to be associated with outcomes via pleiotropic pathways.^[12]^ Moreover, we used the GWAS Catalog to search for a possible pleiotropic SNP in the forward MR analysis. We conducted a series of sensitivity analyses that were robust to horizontal pleiotropy in the reverse MR analysis. However, the forward MR analysis had only 1 or 2 pQTLs as IVs for each protein, making it impossible to conduct the sensitivity analyses.^[42]^ Second, we may have missed some important proteins that could be therapeutic targets for DD because we used only cis-acting pQTLs. Including nonpleiotropic trans-acting pQTLs^[12]^ or using GWAS datasets with a larger sample size could prioritize a larger number of causal proteins. Third, as the present study was based on populations of European ancestry, the results may not be generalizable to other populations. The European have higher rates for DD than the African and Asian,^[43]^ suggesting that different mechanisms may be underlying among ethnic groups. Non-European populations should be included in future studies to validate the findings across different ethnicities. Fourth, to our knowledge, pQTL data are currently available only from blood and brain tissues.^[44]^ Future studies using pQTL data from intestinal tissues may be warranted. However, plasma proteins may originate from almost every tissue in the body as they are secreted into the circulation to perform their functions and mediate their communications with other tissues, or they may leak into the blood due to tissue injury. Therefore, the human plasma proteome may provide an attractive source for elucidating molecular mechanisms of disease at systemic levels.^[45]^ Finally, follow-up functional studies in future are necessary to validate the role of CCN3/NOV protein in the pathogenesis of DD.

In conclusion, we explored potential causal genes for DD for the first time at the protein level and prioritized the CCN3/NOV protein as a protective factor among 735 plasma proteins using MR and Coloc approaches. Although follow-up functional studies are necessary, our study may shed light on the development of a novel therapeutic target for diverticula disease.

## Acknowledgments

We highly appreciate the pQTL and GWAS studies used in the present study.

## Author contributions

**Conceptualization:** Masahiro Yoshikawa.

**Data curation:** Masahiro Yoshikawa.

**Formal analysis:** Masahiro Yoshikawa.

**Investigation:** Masahiro Yoshikawa.

**Methodology:** Masahiro Yoshikawa.

**Project administration:** Masahiro Yoshikawa.

**Resources:** Masahiro Yoshikawa.

**Supervision:** Kensuke Asaba.

**Validation:** Kensuke Asaba.

**Visualization:** Masahiro Yoshikawa.

**Writing – original draft:** Masahiro Yoshikawa.

**Writing – review & editing:** Kensuke Asaba.

## Supplementary Material


